# На пути к надлежащей статистической практике. Валидизированный вопросник CORSTAN для оценки корректности статистического анализа в медицинских исследованиях

**DOI:** 10.14341/probl12797

**Published:** 2021-12-30

**Authors:** О. Ю. Реброва, В. К. Федяева, В. А. Аксёнов

**Affiliations:** Российский национальный исследовательский медицинский университет им. Н.И. Пирогова; Национальный медицинский исследовательский центр эндокринологии; Межрегиональная общественная организация «Общество специалистов доказательной медицины»; Межрегиональная общественная организация «Общество специалистов доказательной медицины»; Межрегиональная общественная организация «Общество специалистов доказательной медицины»

**Keywords:** доказательная медицина, методологическое качество, анализ данных, статистический анализ, ошибки, вопросник, CORSTAN, валидность

## Abstract

**ОБОСНОВАНИЕ:**

ОБОСНОВАНИЕ. В соответствии с концепцией доказательной медицины методологическое качество исследования определяется двумя его аспектами: рисками систематических ошибок и рисками некорректности анализа данных. Минимизация обоих рисков повышает внутреннюю валидность исследования. Существуют многочисленные рекомендации и руководства по проведению статистического анализа данных в медицинских исследованиях и его представлению в публикациях, однако до настоящего времени международным сообществом не был выработан какой-либо формализованный вопросник для оценки качества статистического анализа, который был бы предназначен для рецензентов.

**ЦЕЛЬ:**

ЦЕЛЬ. Разработать инструмент формализованной оценки качества статистического анализа, представленного в научных медицинских публикациях.

**МАТЕРИАЛЫ И МЕТОДЫ:**

МАТЕРИАЛЫ И МЕТОДЫ. Вопросник разработан на основе многолетнего опыта авторов в области статистического анализа данных и рецензирования статистических аспектов статей и диссертаций. При разработке вопросника принимались во внимание рекомендации SAMPL, документ ICH E9 и другие рекомендации. Выполнена внутренняя валидизация вопросника, заключавшаяся в проведении независимой оценки двумя экспертами 20 случайно отобранных в elibrary.ru статей о рандомизированных контролируемых испытаниях (РКИ), и дальнейшем статистическом анализе согласованности заключений экспертов.

**РЕЗУЛЬТАТЫ:**

РЕЗУЛЬТАТЫ. Разработан вопросник CORSTAN (CORrect STatistical ANalysis), состоящий из двух частей: первая часть (10 вопросов) предназначена для оценки исследований любых дизайнов, вторая (8 вопросов) – для дополнительной оценки РКИ. Предложена стратификация риска некорректности статистического анализа. Оценка внутренней валидности вопросника показала ее высокий уровень как в отношении согласованности оценок рецензентов по каждому из вопросов, так и в отношении оценки каждой из статей как по сумме баллов, так и по уровню риска некорректности статистического анализа.

**ЗАКЛЮЧЕНИЕ:**

ЗАКЛЮЧЕНИЕ. Использование вопросника и шкалы позволит упростить и гармонизировать статистическое рецензирование публикаций и рукописей в различных институциях – научных журналах, диссертационных советах и т.д. Вопросник также может быть полезен и авторам в процессе подготовки рукописей, он будет способствовать повышению качества не только публикаций, но и самих исследований. Мы планируем усовершенствовать вопросник по мере накопления опыта его применения.

## Abstract (en)

**BACKGROUND:**

BACKGROUND: In evidence-based medicine, the research methodology is determined by the risks of systematic biases and incorrect data analysis. Minimizing both risks increases the internal validity of the study. There are numerous recommendations and guidelines for data analysis and reporting, but the international community has not yet developed a questionnaire for reviewers to assess the quality of statistical analysis.

**AIM:**

AIM: To develop a tool for formalized assessment of the quality of statistical analysis presented in scientific medical publications.

**MATERIALS AND METHODS:**

MATERIALS AND METHODS: The questionnaire was developed based on the authors' decades of experience in statistical data analysis and reviewing the statistical aspects of biomedical articles and dissertations. The SAMPL guidelines, ICH E9, and other guidelines were taken into account when developing the questionnaire. Internal validation of the questionnaire was based on an independent assessment by two experts of 20 randomly selected articles on randomized controlled trials (RCTs) from elibrary.ru, and further statistical analysis of the agreement of experts' conclusions.

**RESULTS:**

RESULTS: The CORSTAN (CORrect STatistical ANalysis) questionnaire was developed, which consists of two parts: the first part (10 questions) is intended for evaluating studies of any designs, while the second (following eight questions) is for additional assessment of RCTs. A stratification of the risk of incorrect statistical analysis is proposed. The evaluation of the questionnaire's internal validity showed its substantial and almost perfect agreement for each question and each article both in the sum of points and risk level.

**CONCLUSION:**

CONCLUSION: The use of the questionnaire will simplify and harmonize the statistical review of publications and manuscripts in various institutions - scientific journals, dissertation boards, etc. The questionnaire can also be helpful for authors during preparing manuscripts; it will also help improve the quality of publications and research itself. We plan to improve the questionnaire as we gain experience in its application.

## ОБОСНОВАНИЕ

Под надлежащей статистической практикой (Good Statistical Practice, GSP) можно понимать систему (арсенал) принципов, методов и процедур для проведения методически и технически безупречного (корректного) статистического анализа результатов научного исследования и их публикации. Практическое ее использование требует от пользователя обширных знаний и навыков.

В соответствии с концепцией доказательной медицины методологическое качество исследования определяется двумя его аспектами:

Оба риска мы предложили оценивать как низкие, средние или высокие, какие бы системы оценок и шкалы при этом ни использовались, и делать на основании этих оценок совокупное заключение о методологическом качестве исследования [1].

Риск систематических ошибок оценивается по-разному для исследований разных дизайнов. Нами (Ребровой О.Ю. и соавт.) ранее были предложены переводы на русский язык ряда опросников для оценки риска систематических ошибок в исследованиях следующих дизайнов: рандомизированные контролируемые испытания (РКИ) (вопросник Кокрановского сотрудничества Risk-of-Bias), метаанализ (вопросник AMSTAR), сетевой метаанализ (вопросник ISPOR), когортные исследования и исследования «случай-контроль» (вопросник Newcastle-Ottawa), одномоментные исследования диагностических тестов (вопросник QUADAS).

В отношении статистического анализа по-прежнему отмечается существенная обеспокоенность его качеством, и за последние 20 лет в этом отношении мало что изменилось [2]. При рецензировании статей оценка статистического анализа если и выполняется, то рецензентами предметной области (subject reviewer) в рамках своих рецензий. Их знания, к сожалению, обычно совершенно недостаточны для статистического рецензирования.

Существуют рекомендации SAMPL по описанию статистического анализа в статьях [3], их перевод на русский язык [4] и более структурированная версия [5], а также многочисленные чек-листы, предназначенные как для авторов, так и для рецензентов, например [6][7], однако до настоящего времени международным сообществом не выработан какой-либо формализованный вопросник для оценки качества статистического анализа, предназначенный для рецензентов.

## ЦЕЛЬ ИССЛЕДОВАНИЯ

Разработать инструмент формализованной оценки качества статистического анализа, представленного в научных медицинских публикациях.

## МАТЕРИАЛЫ И МЕТОДЫ

Вопросник разработан на основе многолетнего опыта авторов в области статистического анализа данных и рецензирования статистических аспектов статей и диссертаций. При разработке данного вопросника принимались во внимание рекомендации САМПЛ [4] и документ ICH E9 Statistical principles for clinical trials1. Черновик вопросника был ранее включен в ведомственный документ «Методические рекомендации по оценке качества статистического анализа в клинических исследованиях» (ФГБУ «ЦЭККМП» Минздрава России, 2017), однако в настоящее время объем и все пункты этого вопросника переработаны.

Валидизация вопросника выполнялась по случайно отобранным статьям. Для этого в Научной электронной библиотеке (elibrary.ru) 05-01-2021 был выполнен поиск документов по запросу «рандомизированное» с применением следующих фильтров:

Получено 172 статьи, список ранжирован по умолчанию. Из них случайным образом (с применением инструмента https://www.graphpad.com/quickcalcs/randomselect1/) выбраны 20 статей.

Оценка каждой статьи проводилась независимо двумя экспертами. Далее оценивалась внутренняя валидность вопросника следующими способами:

a. согласованность ответов экспертов по каждому вопросу отдельно;

b. различия суммарных баллов двух экспертов;

c. корреляция суммарных баллов двух экспертов;

d. согласованность уровней риска некорректности статистического анализа, оцененных двумя экспертами.

Также оценивалась корреляция суммарных баллов по вопроснику с двухлетними импакт-факторами журналов в РИНЦ на год публикации оцениваемой статьи, их ассоциация с вхождением журнала в перечень Russian Science Citation Index (RSCI) и базу данных Scopus (сведения получены с веб-сайта elibrary.ru).

Для оценки согласованности заключений использовали оценку коэффициента каппа (https://www.graphpad.com/quickcalcs/kappa1/). Интерпретацию коэффициента согласия выполняли в соответствии с [8]. Описательная статистика для сумм баллов представлена медианами и квартилями. Применялись непараметрические методы — тест Вилкоксона, двусторонний точный критерий Фишера (ТКФ2), корреляционный анализ Спирмена. Статистический анализ выполняли в ППП Statistica v. 13 (TIBCO Software Inc., США). Пороговым уровнем значимости считали 0,05.

Для оценки надежности вопросника рассчитывали коэффициент альфа Кронбаха в пакете PASW Statistics (SPSS) v.18 (IBM, США).

## РЕЗУЛЬТАТЫ

В настоящей публикации мы представляем вопросник и шкалу для оценки риска некорректности статистического анализа (табл. 1). Вопросник разделен на две части: первая часть (вопросы №1–10) предназначена для исследований любых дизайнов, вторая (вопросы №11–18) — для дополнительной оценки РКИ. Допустимые ответы на вопросы — «Да» (1 балл), «Нет» (0 баллов). Ситуации неопределенности (условный ответ — «Неясно») следует консервативно трактовать как «Нет». Таким образом, минимальное количество баллов — ноль, максимальное — 18 (для РКИ) и 10 (для исследований других дизайнов).

Принято допущение, что «веса» вопросов равны, однако в дальнейшем это допущение предполагается пересмотреть.

Оценку риска некорректности предлагается стратифицировать следующим образом (табл. 2).

Вопросник назван нами CORSTAN (акроним от CORrect STatistical ANalysis).

В табл. 3 приведены результаты анализа согласованности оценок экспертов по каждому из вопросов. По двум вопросам (№ 6 и 8) расчет коэффициента каппа был невозможен, т.к. дисперсия ответов отсутствовала (все ответы обоих экспертов были «Нет»). По остальным вопросам наблюдалось существенное или отличное согласие.

Медианы и квартили сумм баллов 20 статей были следующими: у первого эксперта — 6,5 [ 4; 9,5 ], у второго эксперта — 6,5 [ 4; 9 ]. Статистически значимых различий оценок экспертов не выявлено (Р=0,343, тест Вилкоксона). Корреляция оценок является сильной (r=0,96; 95% ДИ 0,89–0,98; P<0,001, тест Спирмена).

Отличным также было согласие экспертов в отнесении статей к уровням риска (K=1). Ни одна статья не была отнесена к низкому риску, по двум остальным уровням риска статьи распределились поровну у обоих экспертов.

Не выявлена ассоциация суммарных баллов с двухлетним импакт-фактором журнала в РИНЦ, вхождением журналов в перечень RSCI (12 из 20 статей) и базу данных Scopus (10 из 20 статей). Интересно, что также не выявлена ассоциация между включенностью журналов в RSCI и Scopus (табл. 4).

Показатель надежности (альфа Кронбаха) шкалы вопросника равен 0,629.

**Table:** Таблица 1. Вопросник CORSTAN для оценки риска некорректности статистического анализа в исследованиях по публикациям о них

№	Вопросы и пояснения к ответам
1	Использован ли валидизированный статистический пакет?
Да (1 балл)	Использован валидизированный статистический пакет (SAS, Stata, Statistica, SPSS или др.) или онлайн-калькулятор (со ссылкой на источник)
Нет (0 баллов)	Использованы невалидизированные статистические пакеты (например, собственные разработки) или не приведена информация об используемом программном обеспечении
2	Указан ли пороговый уровень статистической значимости?
Да	Указан, или статистические гипотезы не проверялись
Нет	Не указан
3	Отсутствуют ли ошибки в описательной статистике количественных и качественных признаков?
Да	Ошибки не выявлены
Нет	Нет, например: •неясно, какие именно параметры распределений приведены •для признаков с неизвестным видом распределения приведены среднее и среднеквадратическое отклонение M (SD) •в качестве меры рассеяния приведена стандартная ошибка среднего (SEM, m) •есть ошибки в представлении абсолютных или относительных частот
4	Адекватны ли использованные статистические методы задачам и данным?
Да	Адекватны задачам и данным
Нет	Неадекватны задачам и/или данным или недостаточно информации для оценки
5	Отсутствуют ли ошибки в применении статистических методов?
Да	Ошибки не выявлены
Нет	Выявлены ошибки, например, при проверке гипотез, расчете доверительных интервалов, применении методов, требующих выполнения допущений; невозможно оценить
6	Является ли описание результатов статистического анализа достаточно полным?
Да	Да, является
Нет	Нет, не является, например: •не указаны использованные статистические тесты •не приведена информация о наличии/отсутствии пропусков в данных •не указано число наблюдений в таблицах или на рисунках •недостаточно полно описаны результаты многомерного анализа или анализа времени до события
7	Приведены ли точные рассчитанные уровни статистической значимости р (как минимум два значащих разряда), а не неравенства?
Да	Приведены точные рассчитанные уровни статистической значимости р, или приведены неравенства для некритического диапазона (р>0,05), или проверка статистических гипотез не выполнялась
Нет	Не приведены
8	Применен ли способ преодоления проблемы множественных сравнений?
Да	Применен (например, описано применение поправки для порогового уровня значимости, применены post hoc тесты, применен иерархический подход к проверке гипотез), или проблема множественных сравнений отсутствует
Нет	Не применены или лишь упомянуты способы преодоления проблемы множественных сравнений
9	Приведены ли доверительные интервалы для основных результатов исследования?
Да	Приведены (в РКИ — хотя бы для первичного исхода)
Нет	Не приведены
10	Корректна ли интерпретация результатов статистического анализа в формулировке выводов?
Да	Корректна
Нет	Некорректна или невозможно оценить
Далее только для РКИ
11	Ясна ли проверяемая гипотеза?
Да	Да, например: •явно сформулирована проверяемая гипотеза (превосходства, не меньшей эффективности, эквивалентности) •в исследовании изучали эффективность вмешательства в сравнении с плацебо или отсутствием изучаемого вмешательства, что предполагает проверку гипотезы превосходства •проверяемая гипотеза понятна из способа расчета необходимого объема выборки
Нет	Нет информации
12	Описан ли расчет необходимого объема выборки?
Да	Да, приведена полная информация
Нет	Информация недостаточно полная или отсутствует
13	Если тестируют гипотезу превосходства, то анализировали ли данные пациентов по намерению лечить (intention to treat)? Если тестируют гипотезу не меньшей эффективности или эквивалентности, то анализировали ли данные пациентов, закончивших наблюдение (per protocol)?
Да	Выполнено ли хотя бы одно из следующих условий: •проанализированы данные пациентов по намерению лечить или данные пациентов, получивших хотя бы одну дозу вмешательства (при тестировании гипотезы превосходства) •количество анализируемых пациентов по первичному исходу совпадает с количеством пациентов, распределенных по группам (при тестировании гипотезы превосходства) •проанализированы данные пациентов, которые получили полный курс вмешательства в соответствии с протоколом (при тестировании гипотез не меньшей эффективности или эквивалентности) •количество анализируемых пациентов было меньше, чем количество пациентов, распределенных по группам (при тестировании гипотез не меньшей эффективности или эквивалентности) •для первичного исхода применялся анализ времени до события
Нет	Нет информации об использованном наборе данных или гипотеза не ясна
14	Указан ли метод заполнения пропусков по выбывшим пациентам в анализе набора данных по намерению лечить (ITT)?
Да	Указан метод заполнения пропусков или пропусков не было или анализ ITT не проводился
Нет	Нет информации о пропусках или способе их заполнения
15	Приведены ли частоты и причины выбывания из групп?
Да	Приведены или нет выбывания
Нет	Частоты и/или причины выбывания из групп не приведены или неизвестно, было ли выбывание
16	Приведены ли описательная статистика и результаты статистического сравнения исходных характеристик групп?
Да	Приведены описательная статистика исходных характеристик групп и результаты сопоставления исходных характеристик групп (в виде рассчитанных уровней статистической значимости р) по всем или хотя бы основным характеристикам групп
Нет	Не приведена описательная статистика исходных характеристик групп или исходные характеристики групп не сопоставлены (не приведены рассчитанные уровни статистической значимости р)
17	Проведен ли анализ влияния вмешивающихся факторов (конфаундеров) на величину эффекта?
Да	Анализ проведен или конфаундеры при сопоставлении исходных характеристик групп не выявлены
Нет	Анализ не проведен или не приведены доказательства отсутствия конфаундеров
18	Приведены ли показатели относительного и абсолютного размера эффекта и их доверительные интервалы хотя бы для первичного исхода?
Да	Приведены
Нет	Не приведены

**Table:** Таблица 2. Стратификация риска некорректности статистического анализа по сумме баллов вопросника CORSTAN

Уровень риска	РКИ	Исследования других дизайнов
Низкий	13–18 баллов	8–10 баллов
Средний	7–12 баллов	5–7 баллов
Высокий	0–6 баллов	0–4 балла

**Table:** Таблица 3. Согласованность оценок экспертов

№ вопроса	Каппа	Интерпретация согласия по [15]
1	1	Отличное согласие
2	1	Отличное согласие
3	1	Отличное согласие
4	1	Отличное согласие
5	0,780	Существенное согласие
6	Оценка невозможна (все ответы «Нет»)	Ответы рецензентов полностью совпали
7	1	Отличное согласие
8	Оценка невозможна (все ответы «Нет»)	Ответы рецензентов полностью совпали
9	0,765	Существенное согласие
10	0,800	Существенное согласие
11	1	Отличное согласие
12	1	Отличное согласие
13	0,875	Отличное согласие
14	0,794	Существенное согласие
15	0,765	Существенное согласие
16	1	Отличное согласие
17	0,875	Отличное согласие
18	0,886	Отличное согласие

**Table:** Таблица 4. Включенность в RSCI и Scopus журналов, в которых опубликованы 20 проанализированных статей

RSCI	Scopus
не включен	включен
Не включен	5	3
Включен	5	7
	P=0,650, ТКФ2

## ОБСУЖДЕНИЕ

Предложен вопросник, в котором 10 вопросов применимы для оценки исследований любых дизайнов, а 8 дополнительных вопросов — специально для оценки РКИ. Оценка внутренней валидности по 20 случайно отобранным публикациям показала ее высокий уровень как в отношении согласованности оценок рецензентов по каждому из вопросов, так и в отношении оценки каждой из статей как по сумме баллов, так и по уровню риска некорректности статистического анализа.

Следует отметить, что ни в одной статье не был определен низкий риск некорректности статистического анализа, по остальным двум уровням риска статьи распределились поровну, что свидетельствует о недостаточном качестве статистического анализа в публикуемых исследованиях. Следствием такого распределения суммарных баллов является и полученное нами невысокое значение показателя надежности шкалы. Для улучшения этого показателя требуется целенаправленное включение в анализ статей с высоким качеством статистического анализа, что было невозможно реализовать при случайном отборе статей.

Непосредственной причиной недостаточного качества статистического анализа, по нашему мнению, является отсутствие хотя бы выборочного статистического рецензирования в процессе редакционной подготовки статей к публикации. Более глубокой причиной является недостаток квалифицированных специалистов в области статистического анализа медицинских данных в отечественных исследовательских организациях и вузах.

Попытка внешней валидизации вопросника показала, что качество статистического анализа в публикациях не ассоциировано с оценкой качества журнала при формировании перечня RSCI и включения в базу данных Scopus. Такой результат оказался для нас вполне ожидаемым, однако, как мы полагаем, эту ситуацию следует менять, т.к. риск некорректности результатов статистического анализа определяет методологическое качество статей, что, в свою очередь, может приводить к смещенности результатов и быть одной из важных причин развернувшегося в последние годы кризиса воспроизводимости в биомедицине [9–12].

Надеемся, что использование вопросника и шкалы позволит упростить и гармонизировать статистическое рецензирование публикаций и рукописей в различных институциях — научных журналах, диссертационных советах и т.д. С учетом того, что статистическое рецензирование в большинстве случаев выполняется не специалистами, а рецензентами предметной области, вопросник может быть полезен и им. Конечно, непосредственное использование вопросника неспециалистами может оказаться сложным (в частности, по пунктам 3–6, 10), поэтому мы полагаем, что будет целесообразным проведение тренингов для рецензентов по использованию вопросника, что будет также способствовать более широкому его применению.

Вопросник также может быть полезным и действенным инструментом для выявления и профилактики негативных последствий применения неадекватных статистических процедур и методов. Его использование в процессе предпубликационной активности авторов, рецензентов и редакторов научных изданий будет способствовать повышению качества не только публикаций, но и самих исследований. Вопросник будет усовершенствоваться по мере накопления опыта его применения.

Ограничения исследования

Ограничениями работы следует считать малый объем выборки, валидизацию вопросника только по РКИ (т.е. длинной версии вопросника) и только авторами вопросника, неудачу внешней валидизации вопросника (отметим, что внешняя валидизация была вспомогательной задачей).

Направления дальнейших исследований

Планируется продолжить валидизацию вопросника по РКИ с привлечением внешних экспертов, а также выполнить валидизацию короткой версии вопросника по исследованиям других дизайнов. Также в перспективе возможно уточнение формулировок вопросника, приписывание неравных весов вопросам. Как и другие подобные инструменты, вопросник будет усовершенствоваться, будут выпускаться новые его версии.

## ЗАКЛЮЧЕНИЕ

Предложен вопросник из 18 пунктов, предназначенный для оценки представления процедуры и результатов статистического анализа в публикациях о медицинских исследованиях любых дизайнов. Часть вопросов специально предназначена для оценки РКИ. Вопросник имеет высокую внутреннюю валидность и может быть использован как рецензентами, так и авторами при подготовке рукописей.

## ДОПОЛНИТЕЛЬНАЯ ИНФОРМАЦИЯ

Источники финансирования. Работа выполнена по инициативе авторов без привлечения финансирования.

Конфликт интересов. Авторы декларируют отсутствие явных и потенциальных конфликтов интересов, связанных с содержанием настоящей статьи.

Участие авторов. Реброва О.Ю. — разработка концепции и дизайна исследования, получение и анализ данных, интерпретация результатов, написание статьи; Федяева В.К. — разработка дизайна исследования, интерпретация результатов, редактирование статьи; Аксенов В.А. — получение данных, интерпретация результатов, редактирование статьи. Все авторы одобрили финальную версию статьи перед публикацией, выразили согласие нести ответственность за все аспекты работы, подразумевающую надлежащее изучение и решение вопросов, связанных с точностью или добросовестностью любой части работы.

Благодарности. Авторы признательны к.б.н. Н.Н. Хромову-Борисову и к.т.н. В.П. Леонову за обсуждение вопросника и ценные замечания.

1. https://database.ich.org/sites/default/files/E9_Guideline.pdf

## References

[cit1] Реброва О. Ю. О принципах экспертизы методологического качества оригинальных медицинских исследований // Медицинские технологии. Оценка и выбор. – 2014. - №4. – С. 15-18. ISSN: 2219-0678.

[cit2] Hardwicke TE, Goodman SN. How often do leading biomedical journals use statistical experts to evaluate statistical methods? The results of a survey. PLoS ONE. 2020;15(10):e0239598. DOI 10.1371/journal.pone.0239598. 10.1371/journal.pone.0239598 PMC7529205 33002031

[cit3] Lang T, Altman D. Basic statistical reporting for articles published in clinical medical journals: the SAMPL Guidelines. Int J Nurs Stud. 2015; 52(1):5-9. DOI 10.1016/j.ijnurstu.2014.09.006. 10.1016/j.ijnurstu.2014.09.006 25441757

[cit4] Ланг Т., Альтман Д. Основы описания статистического анализа в статьях, публикуемых в биомедицинских журналах. Руководство «Статистический анализ и методы в публикуемой литературе (САМПЛ)». Медицинские технологии. Оценка и выбор. 2014;1:11-16. ISSN: 2219-0678.

[cit5] Indrayan A. Reporting of Basic Statistical Methods in Biomedical Journals: Improved SAMPL Guidelines. Indian pediatrics. 2020;57:43-48. PMID: 31937697. 31937697

[cit6] Petrovečki M. The role of statistical reviewer in biomedical scientific journal. Biochemia Medica. 2009;19(3):223–30. DOI: 10.11613/BM.2009.020.

[cit7] Greenwood DC, Freeman JV. How to spot a statistical problem: advice for a non-statistical reviewer. BMC Medicine. 2015;13:270. DOI 10.1186/s12916-015-0510-5. 10.1186/s12916-015-0510-5 PMC4629287 26521808

[cit8] Landis JR, Koch GG. The Measurement of Observer Agreement for Categorical Data. Biometrics. 1977;33(1):159-174. doi: https://doi.org/10.2307/2529310 843571

[cit9] Hanin L. Why statistical inference from clinical trials is likely to generate false and irreproducible results. BMC Med Res Methodol. 2017;17(1):127. doi: 10.1186/s12874-017-0399-0. 10.1186/s12874-017-0399-0 PMC5568363 28830371

[cit10] Turkiewicz A, Luta G, Hughes HV, Ranstam J. Statistical mistakes and how to avoid them - lessons learned from the reproducibility crisis. Osteoarthritis Cartilage. 2018;26(11):1409-1411. doi: 10.1016/j.joca.2018.07.017. 10.1016/j.joca.2018.07.017 30096356

[cit11] Bishop D. Rein in the four horsemen of irreproducibility. Nature. 2019;568(7753):435. doi: 10.1038/d41586-019-01307-2. 10.1038/d41586-019-01307-2 31019328

[cit12] Gosselin RD. Statistical Analysis Must Improve to Address the Reproducibility Crisis: The ACcess to Transparent Statistics (ACTS) Call to Action. Bioessays. 2020;42(1):e1900189. doi: 10.1002/bies.201900189. 10.1002/bies.201900189 31755115

